# Prevalence and genotyping of *Pneumocystis jirovecii* in renal transplant recipients—preliminary report

**DOI:** 10.1007/s00436-018-6131-0

**Published:** 2018-11-03

**Authors:** Magdalena Szydłowicz, Katarzyna Jakuszko, Anna Szymczak, Paweł Piesiak, Aneta Kowal, Żaneta Kopacz, Maria Wesołowska, Maria Luísa Lobo, Olga Matos, Andrzej B. Hendrich, Marta Kicia

**Affiliations:** 1 Department of Biology and Medical Parasitology, Wroclaw Medical University, ul. J. Mikulicza-Radeckiego 9, 50-345 Wroclaw, Poland; 2 Department of Nephrology and Transplantation Medicine, Wroclaw Medical University, Wroclaw, Poland; 3 Department of Pulmonology and Lung Cancer, Wroclaw Medical University, Wroclaw, Poland; 4 Global Health and Tropical Medicine, Unit of Medical Parasitology, Instituto de Higiene e Medicina Tropical, Universidade Nova de Lisboa, Lisbon, Portugal

**Keywords:** *Pneumocystis jirovecii*, Renal transplant recipients, Genotyping, Prevalence, Colonization

## Abstract

*Pneumocystis jirovecii* is an opportunistic fungus occurring in human lungs. The group at highest risk consists of HIV-infected and non-HIV-infected immunosuppressed individuals. In these patients, *P. jirovecii* infection may lead to *Pneumocystis* pneumonia; it may, however, persist also in an asymptomatic form. This study aimed to determine the prevalence of *P. jirovecii* and potential risk factors for infection in a group of renal transplant recipients and to characterize the genetic diversity of this fungus in the studied population. Sputum specimens from 72 patients were tested for presence of *P. jirovecii* using immunofluorescence microscopy, as well as nested PCR targeting the *mtLSU* rRNA gene. Genotyping involving analysis of four loci—*mtLSU* rRNA, *CYB*, *DHPS*, and *SOD—*was used to characterize the diversity of the detected organisms. *Pneumocystis* DNA was detected in eight (11.11%) patients. It has been shown that low eosinophil count and dual immunosuppressive treatment combining prednisone and calcineurin inhibitors are potential risk factors for colonization. Analysis of genotype distribution showed an association of the wild-type genotype of *mtLSU* rRNA with lower average age of patients and shorter time after kidney transplantation. Furthermore, *CYB* 2 genotype was detected only in patients with the ongoing prophylaxis regimen. In conclusion, renal transplant recipients are at risk of *Pneumocystis* colonization even a long time after transplantation. The present preliminary study identifies specific polymorphisms that appear to be correlated with certain patient characteristics and highlights the need for deeper investigation of these associations in renal transplant recipients.

## Introduction

*Pneumocystis jirovecii* is a unicellular parasitic fungus occurring in the human lower respiratory tract. Infection with this opportunistic pathogen is a threat for patients with impaired immunity, where it often leads to the development of symptoms of *Pneumocystis pneumonia* (PcP). One of the groups at highest risk of acquiring *P. jirovecii* infection is that of non-HIV-infected patients receiving immunosuppressive treatment, such as transplant recipients. PcP in these patients is characterized by rapid onset and fast progression of severe symptoms, which often lead to death (Roblot et al. 2002; Roux et al. 2014). Widespread use of prophylaxis and treatment for *P. jirovecii* has led to the reduction of the incidence of PcP in the groups at risk, such as renal transplant recipients (RTRs). In some cases, the infection may also remain asymptomatic. This phenomenon, known as colonization, serves as a reservoir of the pathogen in the general population. Persistent fungal colonization may also contribute to the selection of drug-resistant genetic variants, for instance due to the application of specific medications in prophylactic treatment. One of such agents is co-trimoxazole, the first choice for prevention and treatment of *P. jirovecii* infections. This drug is a combination of two components: trimethoprim (TMP) and a sulfa drug, sulfamethoxazole (SMX), active against two enzymes playing a key role in the metabolic pathway of synthesis of folic acid: dihydrofolate reductase (DHFR) and dihydropteroate synthase (DHPS), respectively (Lobo et al. 2013). It has been suggested that the previous use of sulfa drugs, which are also employed in many other disorders, may be associated with the exertion of selective pressure on the emergence of mutations within the DHPS gene (Lane et al. 1997). Moreover, the occurrence of such mutations in *Pneumocystis* organisms isolated from infected patients may be associated with the failure of prophylaxis and/or treatment (Kazanjian et al. 1998). As demonstrated for other microorganisms, such as *Escherichia coli*, *Mycobacterium leprae*, *Plasmodium falciparum*, and *Staphylococcus aureus*, point mutations within genes encoding DHPS are responsible for the resistance to sulfa agents (Dallas et al. 1992; Hampele et al. 1997; Wang et al. 1997; Kai et al. 1999). Similar studies for *P. jirovecii* are difficult to conduct, since this organism cannot be cultured in vitro. Therefore, the gold standard in the investigation of *Pneumocystis* diversity relies on multilocus genotyping—the analysis of at least two genetic markers, thereby providing high sensitivity and accuracy (Matos and Esteves 2010). Maitte et al. (2013) proposed a simplified scheme involving three loci important for *Pneumocystis* survival: mitochondrial large subunit (mtLSU) rRNA, cytochrome b (CYB), and superoxide dismutase (SOD). The occurrence of single nucleotide polymorphisms (SNPs) within these genes may result from the influence of various environmental factors. Concurrent analysis of these three loci has a sufficient discriminatory power for epidemiological investigation of *Pneumocystis* genetic diversity. Moreover, the DHPS locus can also be included in such analysis, especially in patients receiving previous TMP-SMX therapy, as it can provide additional information regarding correlation of mutations within this locus with sulfa drug use and prophylaxis failure. To date, two SNPs in the *Pneumocystis* DHPS gene have been described, occurring at the 165 and 171 nucleotide positions. These point mutations lead to the altered structure of the enzyme, as they cause amino acid substitutions Thr55Ala and Pro57Ser in the DHPS protein, respectively. The reported prevalence of mutations in the considered loci is variable, depending on factors such as the type of the study group, as well as the geographical location and various prophylaxis regimens used in different countries (Huang et al. 2000; Esteves et al. 2008; Siripattanapipong et al. 2008; Dimonte et al. 2013; Suárez et al. 2017).

Renal transplant patients, who are often subjected to a strong, lifelong immunosuppressive anti-rejection regimen, constitute one of the main groups at risk of *Pneumocystis* infection. Therefore, 4-month use of TMP-SMX prophylaxis for these patients is recommended by the Current European Best Practice Guidelines (2002), while the Kidney Disease: Improving Global Outcomes (KDIGO) (2009) guidelines propose the duration of 3 to 6 months after transplantation. Due to the efficiency of the proposed prevention scheme, the occurrence of PcP among RTRs has decreased in recent years. However, the infection in these patients may be triggered by *Pneumocystis* strains resistant to sulfa drugs. Moreover, despite the prophylaxis use, colonization may occur. Such carriage is associated with the risk of development of PcP symptoms, for example in the case of deterioration of the general condition or prophylaxis discontinuation. Therefore, the main aims of our study were to (i) investigate the prevalence and main risk factors for *P. jirovecii* infection in RTRs and (ii) characterize potential associations between demographic and clinical data and distribution of specific SNPs, combining analysis of four independent polymorphic loci: mtLSU rRNA, CYB, SOD, and DHPS. The results of molecular analysis were compared with the data of patients with various pulmonary diseases, for whom the prevalence and SNP distribution were described previously (Sokulska et al. 2017). Such an analysis makes it possible to assess the importance of discrepancies in genotype distribution occurring in different populations of patients at risk of *Pneumocystis* infection residing in the same area.

## Materials and methods

### Patients and specimens

The study included *sputum* samples taken from 72 non-HIV-infected patients after renal transplantation who were under the care of the Department and Clinic of Nephrology and Transplantation Medicine of Wroclaw Medical University (Wroclaw, Poland) between August 2015 and April 2018. There were no other criteria for patients’ participation in the study except being renal transplant recipient. Demographic, clinical, and laboratory data, including age, gender, time after kidney transplantation, type and dose of immunosuppressive drugs, information about ongoing prophylaxis, report of any respiratory symptoms, renal function, results of basic laboratory tests, history of cytomegalovirus (CMV) infection, and co-morbidities (ischemic heart disease, diabetes, hypertension; history of myocardial infarction, stroke, or cancer; thyroid, lung, or liver diseases) were recorded upon inclusion. Fresh sputum samples were treated with 1 M dithiothreitol as a mucolytic agent and centrifuged at 5000 rpm for 20 min*.* The sediment was resuspended in the remnant of supernatant. Smear slides for staining were prepared using 20 μl of cell pellets, followed by fixation with high-quality acetone. The remaining amounts of sputum were frozen at − 20 °C for a maximum of 2 weeks without preservatives before molecular analysis. All individual participants included in the study provided written informed consent for the study participation, approved by the Human Research Ethics Committee of Wroclaw Medical University according to agreement no. KB-160/2017.

### Detection of *P. jirovecii*

Nested polymerase chain reaction (*n*-PCR) was used for molecular detection of *P. jirovecii* in patients’ biological specimens. It was performed using Taq polymerase for amplification of the *mtLSU* rRNA gene of this organism, as described previously (Wakefield et al. 1990; Wakefield 1996). In order to obtain DNA for molecular analysis, sputum specimens were homogenized by bead disruption with a Precellys24 Instrument (Bertin Technologies, France) followed by digestion with proteinase K at 56 °C for 1 h. A QIAamp DNA Mini Kit (QIAGEN, Hilden, Germany) was used for total DNA extraction, according to the manufacturer’s instructions. A sample of human-derived *P. jirovecii* DNA was used as a positive control in each experiment, and a sample of distilled water was included as a negative control to exclude contamination. All manipulations during DNA extraction and amplification were performed in separate areas of the laboratory, in laminar flow cabinets, decontaminated previously by UV light and disinfectants with international aseptic attestations. Disposable pipettes, tubes, and reagent aliquots were used to avoid contamination. The *n*-PCR was repeated three times for each sample, and only those with confirmed *Pneumocystis* DNA presence were considered to be positive*.* Additionally, indirect immunofluorescence (IF) staining (MonoFluo kit *P. jirovecii*; Bio-Rad) was applied. Microscopic examination was performed using × 20 and × 40 dry objectives for the screening of the entire stained region, enabling assessment of fungal burden by scoring the total number of cysts observed (Gill et al. 1987). At least two slides were prepared for each patient sample.

### Case definition

Two conditions had to be met for PcP diagnosis: (i) the occurrence of suitable clinical symptoms and signs: dyspnea, low-grade fever (body temperature between 37.5 and 38.3 °C), unproductive (without mucus or any secretion) cough, and typical radiological findings (the presence of bilateral peripheral interstitial infiltrates and/or ground glass opacities) and (ii) positive results of molecular and microscopic examination of sputum specimens. An IF sample was recognized as negative if no fluorescent cysts were identified on a whole slide; detection of more than five fluorescent cysts was indicative of a positive specimen; and presence of one to five cysts seen on the whole slide was interpreted as an equivocal result, suggesting a colonization case (Gill et al. 1987; Midgley et al. 1991). Molecular detection of *P. jirovecii* DNA in patients without any specific symptoms, with a negative or equivocal IF result, was defined as colonization.

### Multilocus typing

Products of *n*-PCR were visualized by electrophoresis using 1% agarose gel. Bands of the expected size for amplification of the *mtLSU* rRNA locus (260 bp) were purified with the Zymoclean Gel DNA Recovery Kit (Zymo Research, Irvine, CA, USA), and DNA was sequenced bi-directionally by a company offering this service commercially. This enabled confirmation of the presence of *P. jirovecii* DNA in a patient sample, as well as identification of polymorphisms occurring at two informative positions of this locus (85 and 248). All samples positive for *mtLSU* rRNA were additionally subjected to *n*-PCR reactions with specific pairs of primers amplifying the *CYB*, *DHPS*, and *SOD* loci, as described previously (Helweg-Larsen et al. 1999; Costa et al. 2005; Esteves et al. 2010b; Monroy-Vaca et al. 2014). Similarly, bands of expected sizes (620 bp, 186 bp, and 578 bp for *CYB*, *DHPS*, and *SOD*, respectively) were visualized, purified, and sequenced as described above. Polymorphisms at informative positions were analyzed: 279, 348, 516, 547, 566, and 838 for *CYB*; 165 and 171 for *DHPS*; 110 and 215 for *SOD* (Achari et al. 1997; Esteves et al. 2010b; Monroy-Vaca et al. 2014)*.* ChromasPro (version 2.1.1, Technelysium Pty Ltd., Australia) and BLAST software accessible online (https://blast.ncbi.nlm.nih.gov) were used for manual sequence alignment. Results were compared with *P. jirovecii* reference gene sequences (GenBank accession no. M58605.1 for *mtLSU* rRNA; AF321304.1 for *CYB*; JX101868.1 for *DHPS*; and KT592347.1 for *SOD*). Genotypes were determined according to the available nomenclature (Beard et al. 2000; Esteves et al. 2010b; Maitte et al. 2013).

Genotyping of *DHPS* in patients with various pulmonary diseases was also performed, as described above. A sufficient amount of material required as a template for *n*-PCR was available for 15 out of 17 *P. jirovecii-*positive patients’ samples (88%) described previously, including eight patients diagnosed with lung cancer, two with interstitial lung diseases, two with lung nodules in diagnosis, one with bacterial pneumonia, one with chronic obstructive pulmonary disease, and one with Churg-Strauss syndrome (Sokulska et al. 2017).

### Statistical analysis

Categorical variables were compared between *Pneumocystis-*positive and *Pneumocystis*-negative patients using the *χ*^2^ or Fisher’s exact tests. Continuous variables were compared using Student’s *t* test. These statistical tests were also applied for the comparison of the data on the prevalence and genotype distribution between RTRs and previously described patients with various pulmonary diseases (Sokulska et al. 2017). A value of *P <* 0.05 was considered significant.

## Results

### *P. jirovecii* prevalence and patients’ characteristics

Among all renal transplant recipients tested (*n* = 72, including 36 males, 36 females), the mean age was 52.5 ± 13.9 years, range 21–76 years. The mean time after kidney transplantation was 78.7 months, ranging from 5 days to 19 years. Nine patients had undergone the second kidney transplantation, and two had undergone the third transplantation. One patient had undergone both kidney and heart transplantation. Immunosuppressive treatment included prednisone, calcineurin inhibitors (tacrolimus or cyclosporine), proliferation signal inhibitors (sirolimus or everolimus), mycophenolate mofetil, or azathioprine. Forty-four (61.1%) patients were receiving treatment combining three of the above drugs, 25 (34.7%) were receiving a dual combination, and 3 (4.2%) were treated with one type of immunosuppressant only (patients who required dialysis after kidney transplant failure). The routine anti-*Pneumocystis* prophylaxis regimen contained co-trimoxazole at a dose of 480 mg a day for 6 months after transplantation. Among all patients examined, 13 were receiving co-trimoxazole prophylaxis during sputum collection, and one patient was receiving only trimethoprim due to a previous allergic reaction to sulfamethoxazole.

Nested PCR amplifying the *mtLSU* rRNA gene of *P. jirovecii* was positive in eight of the 72 (11.11%) patients’ samples. Three of these eight patients showed symptoms compatible of pneumonia (including low-grade fever, dyspnea, and radiological presentation). In samples from only three patients (37.5%), *P. jirovecii* presence was confirmed by IF staining, and only in one of these cases the number of cysts observed on the whole microscope slide was above five. That was the only case with both a positive result of IF staining and the presence of respiratory symptoms typical for PcP. Anti-*Pneumocystis* therapy was introduced in this patient, in view of PcP suspicion. Due to the patient’s allergy to sulfamethoxazole, it consisted of intravenous clindamycin (900 mg every 8 h on the first day and 600 mg every 6 h afterwards) and primaquine for 29 days. The treatment was completed after the negative result of control PCR analysis and pentamidine was introduced for prevention.

The most important characteristics of *P. jirovecii*-positive and *P. jirovecii*-negative patients are listed in Table 1. A statistically significant correlation with colonization was observed for the employment of a dual immunosuppressive regimen consisting of calcineurin inhibitors and prednisone (*P* = 0.041, Fisher’s exact test). Moreover, the mean eosinophil level was lower in *P. jirovecii-*positive patients, as compared to negative ones (*P* = 0.040, Student’s *t* test). There were no significant differences in results of basic laboratory tests for other parameters, CMV infection, or other co-morbidities.

**Table 1:** Comparison of *Pneumocystis jirovecii*-positive and *Pneumocystis jirovecii*-negative patients’ basic characteristics

	*Pneumocystis jirovecii*		
Characteristic	Positive (*n* = 8)	Negative (*n* = 64)	*P* value
Age in years, mean (range)	55.1 (35–69)	52.2 (21–76)	0.292
Sex			
Male	6 (75)	30 (46.9)	0.260
Female	2 (25)	34 (53.1)	0.260
Time after kidney transplantation in months, mean (range)	68.2 (4.7–142.5)	80 (0.16–225.6)	0.080
Kidney transplantation episode			
First	7 (87.5)	54 (84.4)	1.000
Second	1 (12.5)	8 (12.5)	1.000
Third	–	2 (3.1)	1.000
Symptoms	3 (26.5)	17 (37.5)	0.676
Dyspnea	1 (12.5)	5 (7.8)	0.520
Fever	1 (12.5)	12 (18.75)	1.000
Unproductive cough	1 (12.5)	6 (9.4)	0.578
Ongoing prophylaxis	2 (25)	12 (18.75)	0.648
Immunosuppressive regimen			
CI, prednisone, MMF	2 (25)	37 (57.8)	0.131
CI, prednisone, AZA	–	4 (6.2)	1.000
PSI, prednisone, MMF	–	1 (1.6)	1.000
PSI, prednisone	–	1 (1.6)	1.000
CI, prednisone	5 (62.5)	16 (25)	0.041*
CI, MMF	–	3 (4.7)	0.301
Prednisone	1 (12.5)	2 (3.1)	1.000
Other potential risk factors			
CMV infection	1 (12.5)	10 (15.6)	1.000
Eosinophils/μl, mean (range)^a^	74 (0–250)	272 (0–7200)	0.040*
CRP mg/l, mean (range)^b^	32.1 (0.3–176.3)	42.3 (0.2–468)	0.144

Data represent number (%) unless otherwise indicated

*CI* calcineurin inhibitors, *MMF* mycophenolate mofetil, *AZA* azathioprine, *PSI* proliferation signal inhibitors, *CMV* cytomegalovirus

*P value < 0.05

^a^Standard range 0–6000 eosinophils/μl

^b^Standard range 0–5 mg/l

### Multilocus typing

Only *mtLSU* rRNA was fully genotyped in all analyzed specimens. The ratios of efficient amplification of the other genetic fragments were 50% for *SOD*, 62.5% for *DHPS*, and 87.5% for *CYB* (Table 2). Since multilocus genotype is a combination of at least two loci, such complex analysis was not possible due to incomplete data required. Therefore, single genetic fragments were analyzed individually in order to verify the presence of any statistically significant correlations between SNP distribution and patients’ data.

**Table 2:** Polymorphisms identified at the four studied loci

Locus	Genotype	Number of specimens (%)	Single nucleotide polymorphism (amino acid) position and identity
*mtLSU* rRNA	Genotype 1	4 (50)	85C; 248C
	Genotype 2	1 (12.5)	85A; 248C
	Genotype 3	3 (37.5)	85 T; 248C
*CYB*	*CYB* 1	2 (28.6)	279C(93Ile); 348A(116Gly); 516C(172Ile); 547C(183Leu); 566C(189Ser); 838C(280Leu)
	*CYB* 2	2 (28.6)	279C(93Ile); 348A(116Gly); 516C(172Ile); 547C(183Leu); 566C(189Ser); 838 T(280Phe)
	*CYB* 5	1 (14.3)	279 T(93Ile); 348A(116Gly); 516 T(172Ile); 547C(183Leu); 566C(189Ser); 838C(280Leu)
	*CYB* 7	1 (14.3)	279C(93Ile); 348A(116Gly); 516C(172Ile); 547C(183Leu); 566 T(189Leu); 838C(280Leu)
	*CYB* 8	1 (14.3)	279 T(93Ile); 348A(116Gly); 516C(172Ile); 547C(183Leu); 566C(189Ser); 838C(280Leu)
*SOD*	*SOD* 1	2 (50)	110C; 215 T(41Asp)
	*SOD* 2	2 (50)	110 T; 215C(41Asp)
*DHPS*	Genotype 1	5 (100)	165A(Thr); 171C(Pro)

Non-synonymous mutations are underlined

Three of the five previously described *mtLSU* rRNA genotypes were identified: genotype 1 (wild type) was found in four patients’ samples (50%), while genotypes 2 and 3 were detected in one (12.5%) and three (37.5%) other patients, respectively. The most common *CYB* genotypes were *CYB* 1 and 2, both occurring in two (28.6%) cases. The remaining identified genotypes were *CYB* 5, 7, and 8, occurring in one case each (14.3%). *SOD* polymorphisms were identified in four samples only, half of which referred to wild-type genotype (*SOD* 1), and the other half to *SOD* 2. Finally, *DHPS* typing revealed the presence of genotype 1 (wild-type only) in all five successfully amplified samples.

There were no significant differences in SNP distribution and gender, immunosuppressive regimen, or PcP symptoms. However, it was observed that detection of *CYB* 2 genotype was significantly correlated with the ongoing prophylaxis regimen (*P =* 0.047, Fisher’s exact test). Moreover, both mean age (*P =* 0.033, Student’s *t* test) and time after kidney transplantation (*P =* 0.028, Student’s *t* test) were significantly lower in patients with detected wild-type *mtLSU* rRNA genotype (44.5 years, 58.1 months, respectively) than in those with mutant genotypes (65.8 years, 78.3 months, respectively).

*DHPS* typing in patients with various pulmonary diseases was possible in 53% of available samples. Similarly, only genotype 1 was detected. Comparison of RTRs and patients with various pulmonary diseases (whose data on demographic, prevalence, and genotype distribution were described previously; Sokulska et al. 2017) did not reveal any significant differences in SNP distribution or *P. jirovecii* prevalence.

## Discussion

The aim of this study was to evaluate the prevalence of *Pneumocystis* among RTRs in a single transplant center. These patients represent one of the groups at highest risk of opportunistic infections due to their lifelong immunosuppression. Therefore, potential risk factors for *P. jirovecii* infection were also investigated.

Our findings (11.11% overall prevalence) correspond to other published data based on PCR analysis of respiratory specimens obtained from RTRs, which range between 2.2 and 33.3% (Fritzsche et al. 2013; Borstnar et al. 2013; Maruschke et al. 2014; Lee et al. 2017), depending mainly on the type of specimen and the demographic characteristics of the studied population.

In our study, only one patient met all criteria for PcP diagnosis (positive *n*-PCR and IF staining results, and the presence of suitable respiratory symptoms). In the other seven *n*-PCR-positive patients, only two presented any clinical signs and IF confirmed infection in two other cases. However, the number of cysts observed in these specimens was less than five. All these data suggest that the majority of *P. jirovecii* infections detected in this group of patients referred to colonization. This phenomenon, although asymptomatic, remains an important epidemiological and public health problem, as carriers constitute a source of pathogen transmission in the population (Rivero et al. 2008), and such infection may develop into active PcP if the immune status of the carrier deteriorates. Furthermore, it may contribute to the selection of drug-resistant strains (Walker et al. 1998) or even induce unfavorable changes in lung tissue (Probst et al. 2000).

Generally, it is widely accepted that the immunosuppressive treatment promotes *Pneumocystis* colonization and the susceptibility of a given group of patients may be influenced by specific combinations of medications or their doses (Radisic et al. 2003; Neff et al. 2009; Eitner et al. 2011). *Pneumocystis*-positive and *Pneumocystis*-negative patients participating in our study received the same doses and types of respective immunosuppressive drugs. However, of the potential risk factors, a dual immunosuppressive regimen combining calcineurin inhibitors and prednisone was shown to be correlated with *Pneumocystis* infection. Similar dependency has been suggested by Fritzsche et al. (2013), but in this study, the difference was not significant. These data suggest that specific combinations of immunosuppressive medications may influence the risk of *P. jirovecii* colonization. We have also observed a correlation between *Pneumocystis* infection and the measured number of eosinophils, which was within the normal range in both groups of our study, but it was significantly lower in infected as compared to non-infected patients. One of the roles of eosinophils in immune system is protection against certain parasites, as well as bacterial and viral infections (Rosenberg et al. 2013). A study conducted on a mouse model by Eddens et al. (2015) demonstrated that eosinophil-deficient animals were more susceptible to *Pneumocystis* infection and that these cells exhibited an anti-fungal effect against *P. jirovecii.* In conjunction with these data, our study advocates the assumption that decreased number of eosinophils in blood may be another risk factor for *P. jirovecii* infection.

The second aim of our study was to characterize the detected organisms based on SNPs within specific genes occurring in the studied population, in order to perform a preliminary investigation of *Pneumocystis* genetic diversity. The successful typing of all *Pneumocystis*-positive samples was possible only for the *mtLSU* rRNA gene. Since this is a mitochondrial gene, it occurs in many copies in cell; thus, its amplification is possible even in the case of infection with a very low fungal burden. In our study, we found three different *mtLSU* rRNA genotypes, with genotype 1 observed most frequently. Similarly, it is the most frequent version among HIV-infected and non-HIV-infected patients from other countries, such as Portugal, Spain, or France, despite the location of these countries in a different climate zone (Esteves et al. 2008; Hernández-Hernández et al. 2012). Interestingly, our previous research on patients with various pulmonary diseases revealed that genotype 2 was the most common version of *mtLSU* rRNA, while genotype 3 was the least frequent (Sokulska et al. 2017). These differences suggest that genotype distribution may be associated with factors such as the underlying conditions or lifelong immunosuppression, and particular *Pneumocystis* strains may be representative for a given group at risk.

Analysis of the *mtLSU* rRNA distribution in the studied RTR group has revealed that both mean age and time after kidney transplantation were lower in patients harboring *Pneumocystis* strains with wild type than those with other genotypes. This observation suggests that patients with specific clinical or demographic characteristics are more likely to acquire a *P. jirovecii* infection strain harboring particular mutations. In fact, it has been previously suggested that potential SNPs occurring in given *P. jirovecii* genes may be related to specific patient characteristics, such as clinical data or PcP outcome (Montes-Cano et al. 2004; Esteves et al. 2010a). Our finding is yet further evidence for the hypothesis that patients with particular characteristics may be more often infected by strains with a specific genotype.

Lower efficiency of amplification of three other genetic fragments recorded by us may be associated with the fact that genes such as *SOD* and *DHPS* occur in a single copy in the *Pneumocystis* genome, so it is more difficult to amplify them, especially in case of low fungal burden, typical for colonization (Maitte et al. 2013; Monroy-Vaca et al. 2014). Similarly, *DHPS* and *SOD* amplification also failed in most cases of colonized patients with various pulmonary diseases (Sokulska et al. 2017). Nevertheless, comparison of SNP distribution between these two groups of patients did not reveal any significant differences in any of the four genetic fragments studied. Although we were not able to demonstrate any association of a specific *P. jirovecii* genotype with the characteristics of the population studied, such a possibility cannot be ruled out, as the ratio of successful genotyping of all analyzed fragments was relatively low in both groups.

In the present study, two of the five previously described *SOD* genotypes were identified: *SOD* 1 and *SOD* 2. Similar distribution was observed in our previous report (Sokulska et al. 2017). Likewise, in both groups, the only identified version of *DHPS* was genotype 1. It has been indicated by several studies (Lane et al. 1997; Ponce et al. 2017) that the previous use of sulfa drug prophylaxis may exert selective pressure on *DHPS* and be associated with polymorphisms within this gene, resulting in a reduced response to co-trimoxazole treatment. Our study, however, could not confirm this hypothesis. Despite the fact that RTRs were receiving prophylaxis in the past (or, some of them, at the time of *sputum* collection), in all cases identified, the observed genetic version of *DHPS* was the wild type. Our results are in line with other reports where the *DHPS* genotype distribution was not associated with previous exposure to sulfa drugs (Costa et al. 2003). It should be highlighted, however, that these conclusions are drawn on the basis of genotype distribution in only part of the *P. jirovecii-*positive samples.

The *CYB* gene encodes cytochrome b, the target for another drug used in prophylaxis of *Pneumocystis* infections—atovaquone. As in the case of *DHPS*, previous exposure to this medication may be associated with the occurrence of polymorphisms within this locus (Kazanjian et al. 2001) or even lead to drug resistance (Walker et al. 1998). In this study, the two most common genotypes were *CYB* 1 and *CYB* 2. Interestingly, the occurrence of *CYB* 2 was associated with the application of prophylaxis at the time of sputum collection, since this genotype was detected in the only two patients who were receiving prophylaxis at the time of *Pneumocystis* infection. One of them was receiving co-trimoxazole and the other one TMP alone. Although these medications do not contain atovaquone, this is an intriguing finding, since these patients were infected despite the use of anti-*Pneumocystis* prevention. This may be due to the fact that these patients could have acquired the infection with the strain already harboring this genotypic version, which is somehow stronger and more capable of survival in the host, even despite the ongoing action of the prophylactic agents. In fact, one of the samples with *CYB* 2 identified was derived from a patient diagnosed with PcP. Another explanation of such distribution may be the influence of other medication used in these patients on the development of certain polymorphisms, especially that non-synonymous mutations detected in our samples (genotypes *CYB* 2 and *CYB* 7, Table 2) do not concern the atovaquone-binding site of cytochrome b (Walker et al. 1998). Nevertheless, since this observation concerns only two patients, further research should be carried out with a larger study group.

Our results of preliminary research on genetic diversity among *P. jirovecii* occurring in renal transplant recipients indicate the need for further investigation on this issue. A study with a larger sample size, preferably involving specimens from different hospitals in different parts of the same country, should be carried out. According to the data presented here, such complex analysis could reveal interesting associations in SNP distribution.

In conclusion, although the prevalence of 11.11% observed in this study is not very high, it shows that lifelong immunosuppression maintains patients after kidney transplantation in the group at risk of *Pneumocystis* infection. RTRs can be infected even despite the ongoing prophylaxis and regardless of the time that has passed after transplantation surgery. It is necessary to constantly monitor these patients, since in general, symptoms of PcP in non-HIV-infected individuals have a rapid onset and are more severe than in HIV-infected ones, with a higher mortality rate (Roblot et al. 2002). Furthermore, the preliminary genotyping results of this study show that there may be various correlations of distribution of specific SNPs with patients’ clinical data, making some individuals more vulnerable to infection with a *Pneumocystis* strain harboring a given genotype. This hypothesis, however, requires further validation.
